# Differences in Quality of Life, Anxiety and Depression in Patients with Paroxysmal Atrial Fibrillation and Common Forms of Atrioventricular Reentry Supraventricular Tachycardias

**DOI:** 10.1016/s0972-6292(16)30796-3

**Published:** 2014-10-06

**Authors:** Louiza Lioni, Konstantinos Vlachos, Konstantinos P Letsas, Michael Efremidis, Dimitrios Karlis, Dimitrios Asvestas, Vasilios Kareliotis, Sotirios Xydonas, Nikolaos Dimopoulos, Panagiotis Korantzopoulos, Athanasios Trikas, Antonios Sideris

**Affiliations:** 1 Second Department of Cardiology, Laboratory of Cardiac Electrophysiology, "Evangelismos" General Hospital of Athens, Greece; 2 "Dromokaiteio" Psychiatric Hospital of Attica, Greece; 3 Department of Cardiology, University Hospital of Ioannina, Greece; 4 Department of Cardiology, "ELPIS" General Hospital of Athens, Greece

**Keywords:** Quality of life, anxiety, depression, supraventricular tachycardias, atrial fibrillation

## Abstract

**Introduction:**

The aim of this study was to evaluate the differences in quality of life and psychosocial stress parameters among patients with paroxysmal atrial fibrillation (AF) and common forms of atrioventricular reentry supraventricular tachycardias (SVTs).

**Methods and Results:**

The total study population included 106 patients, 54 patients with paroxysmal AF (32 males, age 56.64±12.50 years) and 52 with SVTs (25 males, age 40.46±14.96 years). General health (p<0.01), physical function (p=0.004), role emotion (p=0.002) and role physical (p<0.01) scores were lower in patients who suffered AF. SF-36 physical and mental health summary measures were also significantly lower in the AF group compared to those in SVT group (p<0.01 and p=0.001, respectively). Lower SF-36 total score was observed in patients with AF compared to those with SVTs (p<0.01). Comparing the anxiety and depression scores all the values were higher in patients with AF. Higher STAI-state scores (p<0.01), STAI-trait scores (p=0.039) and BDI scores (p=0.077) were seen in patients who suffered AF comparing to those with SVTs.

**Conclusions:**

Quality of life is significantly impaired and the level of anxiety is significantly higher in patients with AF comparing to those with common forms of SVTs.

## Introduction

Atrial Fibrillation (AF) is associated with a more severe impairment in quality of life (QoL) compared to general population [1]. AF imposes a significant psychosocial burden including depression and anxiety in approximately one third of patients [2]. Psychosocial stress can be elicited by AF episodes and might also predispose to AF initiation. The Framingham Offspring Study demonstrated that tension was an independent predictor of coronary heart disease, AF and mortality in men [3]. Several studies have demonstrated an improvement in QoL after left atrial catheter ablation of paroxysmal AF [4]. Atrioventricular nodal reentry tachycardia (AVNRT) and atrioventricular reentry tachycardia via an accessory pathway (AVRT) are most common types of regular supraventricular tachycardias (SVTs). Due to the paroxysmal nature of the disease, the QoL is increasingly impaired over time [5,6]. SVTs are associated with anxiety in approximately 20% of patients [7]. Patients with SVTs treated with catheter ablation show significant improvement in physical, emotional and social indexes of their health-related QoL [8] and reduction in anxiety symptoms [9]. The aim of this study was to evaluate the differences in QoL and psychosocial stress parameters among patients with paroxysmal AF and common forms of SVT.

## Methods

### Patients

The study population consisted of consecutive patients with symptomatic, drug-refractory paroxysmal AF or symptomatic SVTs (AVNRT or AVRT) who referred for catheter ablation. Patients were classified as having paroxysmal AF according to the current guidelines [10]. Exclusion criteria were age <18years, presence of physical and/or mental insufficiency, left atrial diameter >50mm, intracardiac thrombi documented by transesophageal echocardiography, systolic heart failure [left ventricular ejection fraction (LVEF) <45%, NYHA III-IV], previous ablation for AF, persistent AF, inadequate follow-up and/or inability to provide informed consent. Demographic and clinical characteristics were collected from all patients. Transthoracic and transeshophageal echocardiography was performed in all subjects. LVEF were recorded in all patients. The study protocol was approved by the hospital's Ethics Committee and written informed consent was obtained from all patients.

### Catheter ablation procedures

Left atrial ablation for paroxysmal AF has been described in details elsewhere [11]. In brief, following the transseptal puncture, the three-dimensional geometry of the left atrium was reconstructed using the CARTO 3 system (Biosense Webster, Inc., Diamond Bar, Calif., USA) for isolation of large atrial areas around both ipsilateral pulmonary veins with a 3.5-mm-tip ablation catheter (Thermo Cool Navi-Star, Biosense Webster, Inc., Diamond Bar, Calif., USA). The power settings of the energy were individualized depending on the ablation sites. The end point of electrical pulmonary antral isolation was the absence or dissociation of potentials in the isolated area as documented with the circular mapping catheter (Lasso, Biosense Webster, Inc., Diamond Bar, Calif., USA). The diagnosis of common type AVNRT and AVRT was established using common criteria and diagnostic manoeuvres [12]. The ablation procedure for both SVTs has been previously described [13,14]. Left-sided accessory pathways were mapped using the transaotric approach via the femoral artery under systemic anticoagulation.

### Evaluation of QoL, anxiety and depression

All patients were evaluated for QoL, anxiety and depression using specific questionnaires 24h before ablation. The SF-36 is a multipurpose, short-form health survey with 36 questions that has been used widely to assess QoL in patients with cardiovascular disease [15]. It contains one multi-item scale that assesses 8 individuals health concepts: physical function, role physical, bodily pain, general health, vitality, social function, role emotional, and mental health. Scores in each domain are standardized, ranging from 0 (worst health) to 100 (best health). Physical and mental health summary measures were also assessed on a raw scale of 0 to 100. The SF-36 total score was additionally calculated [16].

The State-Trait Anxiety Inventory (STAI) [17] was used to assess the current level of anxiety and anxiety predisposition. The STAI comprises of two separate self-report scales each containing 20 questions, the first of which evaluates how the patients 'currently feel' (state anxiety) while the second how they 'generally feel' (trait anxiety). The total score on both subscales ranges from 20 to 80, with higher scores indicating greater levels of anxiety. Scores of more than 40 on either subscale were used to indicate significant symptoms of anxiety, as mentioned previously [2,18] Both scales have acceptable internal consistency, with Cronbach's α coefficients of .92 and .90 for the state and trait scales, respectively. There is preliminary evidence showing that the reliability and the validity of the Greek translation of the STAI questionnaire are generally similar to those reported in the international literature [19].

Depression was diagnosed and its severity quantified using the Beck Depression Inventory (BDI) that is commonly used worldwide [20,21]. Various translations of the BDI have been published and this scale was proved to be psychometrically strong and appropriate for use. A Greek version has been applied to neurological patients [22]. The BDI consists of 21 items rated 0 to 3 depending on severity of symptoms. For each item, patients selected one answer that best described their condition during the last week preceding the evaluation. Different questions of BDI evaluate mood, pessimism, past failures, loss of satisfaction, guilt, punishment feelings, self-dislike, suicidal thoughts or wishes, crying irritability, social isolation, perception of own body, difficulties at work, sleeplessness, loss of energy, loss of appetite, body mass reduction and somatic complaints. Questions 1 to 13 evaluate psychosomatic status, while questions 14 to 21 evaluate mental status. The overall score indicates depression severity [23]. Depending on the population, different cut-off points may be applicable. The optimal cut-off points of Greek translation of BDI-21 for clinical and research use were 14/15 [22].

### Statistical Analysis

Continuous variables are expressed as mean ± SD. Categorical variables are presented as absolute numbers and frequencies. Comparisons of the continuous variables between study groups were performed using the Student's t-test. Comparison between categorical variables was performed using the Fisher's exact test. Comparisons of the continuous variables regarding QoL parameters and psychosocial scores were performed using the paired t-test. All reported p values are based on two-sided tests and compared to a significance level of 0.05. The statistical analysis was performed using the SPSS software (version 17.0; SPSS Inc., Chicago, Illinois).

## Results

The total study population included 106 patients, 54 patients with paroxysmal AF (32 males, age 56.64± 12.50 years) and 52 with SVTs (25 males, age 40.46± 14.96 years). Based on the electrophysiological findings, 31 out of 52 patients with SVT displayed AVNRT (59.61%), and 21 patients AVRT (40.38%). As shown in Table 1, patients with SVTs were younger (p<0.01) and displayed better left ventricular systolic function (p=0.015) comparing to those with AF. The duration of arrhythmic episodes were significantly longer in patients with AF in relation to those with SVTs (p<0.01). Patients with AF were more likely to receive class I and class III antiarrhythmic drugs (p<0.01), while there were no significant differences regarding treatment with β-blockers between study groups.

General health (p<0.01), physical function (p=0.004), role emotion (p=0.002) and role physical (p<0.01) scores were lower in patients who suffered AF. SF-36 physical and mental health summary measures were also significantly lower in the AF group compared to those in the SVT group (p<0.01 and p=0.001, respectively). Lower SF-36 total score was observed in patients with AF compared to those with SVTs (p<0.01).

Anxiety scores were higher in patients with AF. Higher STAI-state scores (p<0.01) and STAI-trait scores (p=0.039) were seen in patients who suffered AF comparing to those with SVTs. Depression scores (BDI) were also higher in patients with AF, but not statistically significant comparing to those with SVTs (p=0.077).

## Discussion

The main findings of the present study were: a) QoL parameters were significantly impaired in patients with AF comparing to those suffering regular SVTs; and b) the level of anxiety, depicting psychosocial stress, was significantly higher in patients with AF in relation to those with SVTs.

Several studies have investigated the relationship between psychological variables and arrhythmias. Patients diagnosed with AF experience more psychological distress than healthy individuals. It seems that AF symptom severity deteriorates QoL, and AF recurrence correlates with psychological distress in the form of depression and anxiety [24]. Anger, hostility and tension have been also recognized as risk factors for AF in males [3]. Tension in men and anxiety in both sexes were associated with increased mortality after risk factors for ischemic heart disease had been adjusted [3]. Despite the fact that it remains unclear how acute psychological disturbances lead to AF, some possible mechanisms have been proposed. Previous studies have shown that chronic stress activates the inflammatory cascade [25-30]. The development and maintenance of AF have been associated with systemic inflammation [29,30]. Another potential pathogenetic link between psychological stress and AF recurrence is the activation of the sympathetic system [31-34].

Patients with common regular SVTs (AVNRT or AVRT) are relatively young and otherwise healthy individuals. The hemodynamic consequences are similar in both of these SVTs, which include the shortening of the diastolic filling time of the ventricles, and varying degrees of impairment of synchronization of diastolic filling times of the ventricles and ventricular contraction. This may result in palpitations, dyspnea, hyperventilation, dizziness, sweating, chest pain and anxiety [7]. Palpitations caused by these SVTs are associated with anxiety in approximately 20% of patients and may therefore be misdiagnosed as panic disorder [7]. Due to the paroxysmal character of the tachycardia, with sudden unexpected onset of symptoms, patients are limited in their daily life concerning work, social events and sports [5,6]. Recurrent tachycardia with its unpredictable nature can have a significant impact on a person's entire life situation. The severity of arrhythmia symptoms, ranging from mild palpitations to complete syncope, may not only affect patients during the attack, but also influences their life situation between recurrences impacting quality of life. Frequent attacks are known to have considerable effects on the QoL [5,6]. In our study, the significantly longer duration of arrhythmic episodes in AF patients is possibly implicated in the deterioration of QoL as well as in the induction of psychosocial stress. Furthermore, patients with AF were more commonly under antiarrhythmic drug treatment, a fact that may also have an impact on QoL scores.

## Limitations

This study has some potential limitations. First, a small number of patients were studied in both groups. Second, patients with AF were older, a fact that can cause sizeable differences in QoL and anxiety/depression scales. Third, the frequency and the intensity of the episodes were not evaluated. Finally, data regarding QoL and psychosocial stress parameters following catheter ablation were not assessed.

## Conclusions

Assessing QoL status and psychosocial burden including depression and anxiety in patients with AF and SVTs is an important way to evaluate and describe patients' life situation. Patients with AF manifested significantly lower QoL and higher anxiety levels compared to those with other SVTs. Arrhythmia treatment including catheter ablation is required for patients with AF displaying poor QoL and psychosocial stress.

## Figures and Tables

**Table 1:**
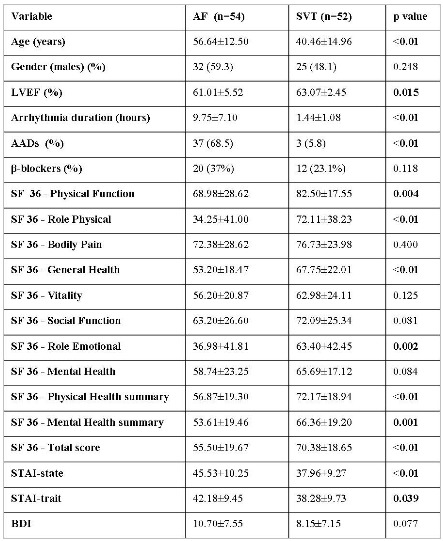
Demographic, echocardiographic, QoL, anxiety and depression data between patients with AF and SVT.

AF: atrial fibrillation; AADs: antiarrhythmic drugs; SVT: supraventricular tachycardia; LVEF: left ventricular ejection fraction; AADs: antiarrhythmic drugs; SF-36: the Short-Form life survey-36 items; STAI: State-Trait Anxiety Inventory; BDI: beck depression inventory.
